# Realistic simulation and experiment reveals the importance of scatterer microstructure in optical coherence tomography image formation

**DOI:** 10.1364/BOE.9.003122

**Published:** 2018-06-13

**Authors:** Paweł Ossowski, Andrea Curatolo, David D. Sampson, Peter R. T. Munro

**Affiliations:** 1 Institute of Physics, Faculty of Physics, Astronomy and Informatics, Nicolaus Copernicus University Grudziadzka 5, 87-100 Torun, Poland; 2 Current address: Instituto de Optica “Daza de Valdés”, Consejo Superior de Investigaciones Cientifícas (IO, CSIC), Madrid, Spain; 3 University of Surrey, Guildford GU2 7XH, United Kingdom; 4 Optical + Biomedical Engineering Laboratory, School of Electrical, Electronic and Computer Engineering, The University of Western Australia, Perth, Western Australia 6009, Australia; 5 Department of Medical Physics and Biomedical Engineering, University College London, Gower Street, London WC1E 6BT, United Kingdom; 6 School of Electrical, Electronic & Computer Engineering, The University of Western Australia, Perth, Western Australia 6009, Australia

**Keywords:** (170.3660) Light propagation in tissues, (050.1755) Computational electromagnetic methods, (180.0180) Microscopy, (110.4500) Optical coherence tomography

## Abstract

Realistic simulation of image formation in optical coherence tomography, based on Maxwell’s equations, has recently been demonstrated for sample volumes of practical significance. Yet, there remains a limitation whereby reducing the size of cells used to construct a computational grid, thus allowing for a more realistic representation of scatterer microstructure, necessarily reduces the overall sample size that can be modelled. This is a significant problem since, as is well known, the microstructure of a scatterer significantly influences its scattering properties. Here we demonstrate that an optimized scatterer design can overcome this problem resulting in good agreement between simulated and experimental images for a structured phantom. This approach to OCT image simulation allows for image formation for biological tissues to be simulated with unprecedented realism.

## 1. Introduction

Full wave modelling of image formation in optical coherence tomography (OCT) has recently become feasible due to advances in both algorithms and computer hardware. Some approaches restrict samples to simple, isolated scatterers, for which the scattered field has an analytic solution. Models for calculating the image of a sphere [1] and cylinder [2] have been recently demonstrated. Simulations for samples with a general refractive index structure require the use of numerical methods. Models employing the finite-difference time-domain (FDTD) method in two-dimensions [3] and the pseudospectral time-domain (PSTD) method in three-dimensions [1] have also been recently demonstrated. More recently, a solution employing a Born series approach has also been demonstrated [4].

A variety of less computationally expensive OCT image formation models have previously been developed. We have reviewed these in detail in previous publications [1,3] and so give only a brief description of these here. Existing models tend to fall into one of two categories: wave optics (see for example [5–10]) and Monte-Carlo based (see for example [11–16]). Up until now, wave optics models have not been full wave and have thus been unable to treat phenomena like multiple scattering, the change in coherence of light due to propagation in tissue and the explicit interference of sample and reference light for deterministic samples. Monte-Carlo models are also not applicable to deterministic refractive index distributions and do not naturally include phenomena such as polarisation, coherence and interference. We have developed our full wave model to address questions which these existing models are unable to answer.

Previous full wave modelling of OCT imaging of blood cells, flowing through a microfluidic channel, suspended above a highly scattering medium is an example where full wave modelling is essential to model image formation. This study revealed that the design of scatterer microstructure significantly impacts upon OCT image formation [17]. In particular, when scatterers are to be represented on a grid with a cell size of length scale comparable to the scatterer itself, the discrete approximation to the scatterer can have scattering properties quite different from the scatterers to be emulated. For example, some simulation results were recently reported where spherical TiO_2_ scatterers of diameter 350nm were required to be simulated using cubes of side 131.7nm [17], resulting in an extremely crude stair-case approximation to the spherical scatterer. It was found that the scattering cross-section of the discrete scatterer differed significantly from that of the spherical scatterer as predicted using Mie theory. A more realistic scatterer design was found that resulted in the same scattering cross-section as the sphere, however, the angular distribution of light scattered for an incident plane still varied significantly between the two cases. This poor representation of scatterer microstructure led to significant divergence between simulated and experimental results.

Motivated by this recent work, we have developed a method for designing discrete scatterers which accurately represent the three-dimensional light scattering properties of the physical scatterers to be modelled, not simply the scattering cross-section. Furthermore, we achieve this across the spectrum of the OCT system, not just at the central wavelength. We also use two well characterised phantoms and a well characterised spectral domain OCT system to demonstrate that close agreement between simulation and experiment can be obtained by using rigorous simulation combined with accurately represented scatterers. In the remainder of this paper we introduce the image formation model and the two physical phantoms used to generate the images in this paper. We then give some examples which demonstrate the problem of representing micro-scale scatterers on a discrete grid before presenting our approach to optimized scatterer design which overcomes this problem. We then perform quantitative comparisons between simulated and experimentally acquired images.

## 2. Image formation model

**Fig. 1:**
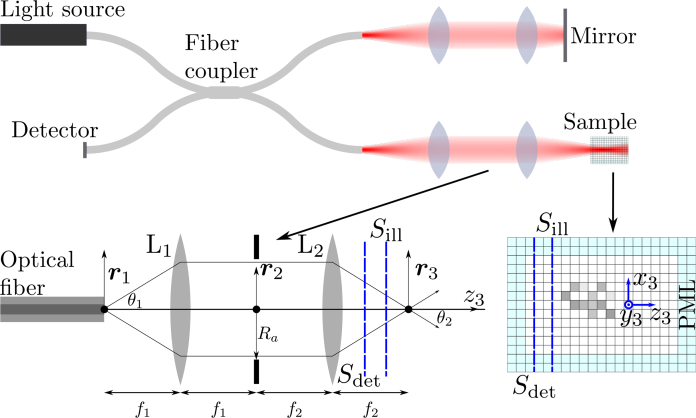
Schematic diagram of the modelled OCT system and the model itself. *S*_ill_ and *S*_det_ represent the planar surfaces upon which the illumination is introduced and the scattered field is detected, respectively. Scattering in free space is simulated by using a perfectly matched layer (PML) which absorbs incident radiation with very low reflection.

The image formation model used in this paper is based on a three-dimensional numerical solution to Maxwell’s equations [1], combined with vectorial theory for light focussing [18] and a computationally efficient method for performing coherent detection of scattered light [19]. The model has been explained previously [1], however, we give a brief outline here for completeness. A schematic diagram of modelled system, and the model itself, is shown in Fig. 1. Although we depict a fibre-based system here, the model may also be applied to free-space systems.

Light detected from both the sample and reference arms is recorded as a function of wavenumber and so the model is equally applicable to both spectral domain and swept source OCT systems. The model can be tailored to either of these systems, however, this was not necessary for the present work. The PSTD method for calculating light scattering by arbitrary refractive index distributions lies at the heart of the model and is depicted in the expanded view of the sample in Fig. 1. The focussed illumination is launched from a plane within the computational grid labelled *S*_ill_ in Fig. 1. Samples are modelled by setting the refractive index of each of the individual cubic cells which make up the computational grid, i.e., the refractive index within each cell is assumed to be uniform. The PSTD algorithm calculates how the focussed illumination is scattered by the sample and the scattered light is recorded at a plane *S*_det_ within the computational grid. For computational efficiency, only the coupling of the scattered field into the optical fiber is recorded, as a function of wavenumber, rather than the field on the entire plane *S*_det_. The focussing theory underlying the imaging model requires that the planes *S*_ill_ and *S*_det_ should be located in homogeneous space above the sample. In particular, there should be at least one plane of homogeneous cells between both planes and any inhomogeneity in the computational grid closest to the planes.

Once the PSTD simulation has completed, the scattered field coupled into the detection fiber is known for all wavenumbers of interest, and, for one scan position of the illumination beam relative to the sample. A similar PSTD simulation is performed to calculate the reference light coupled into the fiber. This allows the light from the sample and reference arms to be interfered and the simulated A-scan to be reconstructed using a discrete Fourier transform.

### 2.1. Illumination

The focussed illumination is introduced into the PSTD computational grid at the plane *S*_ill_ depicted in Fig. 1 using a source condition specifically developed for this purpose [20]. We assume the combination of optical fiber and focussing optics can be represented as a 4f system as shown in Fig. 1, which whilst being an approximation, is a satisfactory one for the purposes of this study. The vectorial form of the focussed field is calculated using the Debye-Wolf integral [18] by making the Gaussian approximation for weakly guiding optical fibers. In particular, it is assumed that a Gaussian beam exits the optical fiber with 1/*e*^2^ width equal to the fiber’s mode field diameter. The only assumption made regarding the polarization state of light exiting the fiber is that it is polarised in the plane perpendicular to the optical axis. The field exiting the fiber, at the central wavenumber (*k*_0_) of the source, is propagated to the focal plane common to lenses L_1_ and L_2_ in Fig. 1 using Fourier optics. The electromagnetic field on plane *S*_ill_ can then be found directly using Debye-Wolf integral which we denote ***e***_ill_(***r***_3_, *k*_0_). This field is used to introduce the focussed field into the PSTD simulation via a time-varying magnetic current density, located on *S*_ill_, of the form:

(1) $$J^{*}(t)=ℜ\left\{-\hat{k}\times e_{\text{ill}}(r_{3},k_{0})\text{exp}(-i\omega_{0}(t-t_{0}))\text{exp}\left(-\pi {\left((t-t_{0})/W\right)}^{2}\right)\right\},$$

where *ω*_0_ = *c*/*k*_0_, *c* is the speed of light in the region where *S*_ill_ is located, *t*_0_ is set so that J^*^(*t*) has a negligible magnitude at *t* = 0 (the starting time of the PSTD simulation), *W* controls the spectral width of the source, and ***k̂*** is the unit vector parallel to the *z*_3_ axis in Fig. 1.

### 2.2. Light sample interaction

The interaction of the focussed illumination with the sample is evaluated using the PSTD method which can be understood by considering a one-dimensional form of Maxwell’s equations:

(2) $$\frac{\partial H_{y}}{\partial t}=\frac{1}{\mu }\left[-\frac{\partial E_{x}}{\partial z}-J_{y}^{*}\right]\;\frac{\partial E_{x}}{\partial t}=-\frac{1}{∊}\frac{\partial H_{y}}{\partial z},$$

where *∊* and *μ* are the electromagnetic permittivity and permeability, respectively. Note that *H_y_* and *E_x_*, in general, vary with *t* and *z* whilst we assume that *μ* and *∊* vary with *z*, however we do not explicitly write this for brevity. Considering the fields at some instant of time, *τ*, the first principle definition of differentiation allows the temporal derivative of the first equation in Eq. (2) to be expanded as:

(3) $$\frac{{H_{y}|}^{\tau +\Delta t/2}-{H_{y}|}^{\tau -\Delta t/2}}{\Delta t}=\frac{1}{\mu }{\left[-\frac{\partial E_{x}}{\partial z}-J_{y}^{*}\right]|}^{\tau }\Rightarrow {H_{y}|}^{\tau +\Delta t/2}=\frac{\Delta t}{\mu }{\left[-\frac{\partial E_{x}}{\partial z}-J_{y}^{*}\right]|}^{\tau }+{H_{y}|}^{\tau -\Delta t/2},$$

and similarly for the second equation in Eq. (2). Equation (3) thus shows how knowledge of the spatial derivative of *E_x_* at time *τ* and *H_y_* at time *τ* − Δ*t*/2, allows *H_y_* to be calculated at a time in the future, *τ* + Δ*t*/2. Manipulation of the second equation in Eq. (2) in a similar way results in a complementary equation for advancing *E_x_*. The defining feature of the PSTD method is that the spatial derivatives of *E_x_* and *H_y_* are calculated from the discretely sampled data using discrete Fourier transforms. This, whilst requiring the fields to be sampled at the Nyquist rate, allows for far more sparse sampling than would be possible in other techniques such as the FDTD method that calculates spatial derivatives using central differences. The PSTD method results in the field ***e***_tot_(***r***_3_, *z_s_*, *t*) throughout the computational grid, which is the summation of the illumination field ***e***_ill_(***r***_3_, *z_s_*, *t*) and scattered field ***e***_sca_(***r***_3_, *z_s_*, *t*) defined as ***e***_sca_ = ***e***_tot_ − ***e***_ill_. Each field quantity can also be evaluated in the spectral domain by applying a discrete Fourier transform at the wavenumbers of interest.

### 2.3. Detection of scattered light

Whilst the scattered light at wavenumber *k*, ***e***_sca_(***r***_3_, *z_s_*, *k*), can be rigorously propagated from the sample space to the optical fiber [21], a more computationally efficient approach is employed in this work. In particular, it can be shown that the coupling of scattered light into the optical fiber can be evaluated within the scalar approximation as:

(4) $$\alpha_{\text{tot}}(k)=\iint_{S_{\text{det}}}{\left(T\;e_{\text{ill}}(r_{3},z_{\text{det}},k)\right)}^{⊤}\;\left(T\;e_{\text{tot}}(r_{3},z_{\text{det}},k)\right){\text{d}}^{2}r_{3}$$

where *T* = [***î***|***ĵ***|**0]**, ^⊤^ is the transpose operator and ***î*** and ***ĵ*** are the unit vectors parallel to the *x*- and *y*-axes, respectively. Equation (4) is able to be calculated as the PSTD algorithm progresses, by evaluating a term contributing to the discrete Fourier transform, with respect to time, of (*T* ***e***_ill_(***r***_3_, *z*_det_, *t*))^⊤^ (*T* ***e***_tot_(***r***_3_, *z*_det_, *t*)) at the conclusion of each PSTD iteration. The PSTD simulation employed a time step of 0.252fs and performed 12000 iterations meaning that light propagation over a time period of 3.03ps was simulated. Free-space systems can be modelled by propagating ***e***_sca_(***r***_3_, *z_s_*, *k*) to the detector at the conclusion of the PSTD simulation. Note that the detector would usually be located where the optical fibre is depicted in Fig. 1.

### 2.4. Noise model

We assume that shot noise is the dominant source of noise in the image. Shot noise was applied at the part of the model where the spectrometer or photodiode is assumed to detect the light incident upon it. In particular, once *α*_tot_(*k*) is calculated for a particular sample refractive index structure, an additional quantity, *α*_ref_(*k*), must also be calculated, which is calculated in the same way as *α*_tot_(*k*) but with a mirror as the sample. The optical power guided by the optical fiber leading to the detector is then described by:

(5) $$i(k)\propto {\left|\alpha_{\text{tot}}(k)-\alpha_{\text{ill}}(k)+\alpha_{\text{ref}}(k)-\alpha_{\text{ill}}(k)\right|}^{2},$$

where *α*_ill_(*k*) is evaluated in the same manner as *α*_tot_(*k*), but with a homogeneous computational grid. It is necessary to subtract the contribution of the illumination from both the scattered and reference fields since the source plane emits a field in both the positive and negative *z*_3_ directions. The number of photons detected within a spectral band centred on wavenumber *k* can then be written as:

(6) n(k)=ηi(k),

where *η* is an effective quantum efficiency. When shot noise is considered, the number of photons detected within a given spectral range is a Poisson random variable with mean *n*(*k*). Noise is thus added by replacing each value *n*(*k*) with a value *ñ*(*k*) which is acquired using a random number generator which takes values from a Poisson distribution with mean *n*(*k*). The value of *η* is chosen in such a way that the signal to noise ratios, for a region of interest in both the simulated and experimental images, agree.

### 2.5. Reconstruction of OCT A-scan

Once noise has been incorporated into the detector measurement, the OCT A-scan can be reconstructed from the spectrally resolved measurements according to:

(7) $$A(z_{3}-z_{\mathrm{ref}})=\frac{1}{2\pi }\int_{0}^{\infty }S(k)\tilde{n}(k)\text{exp}(ik2(z_{3}-z_{\mathrm{ref}}))\text{d}k,$$

where *S*(*k*) is the effective system spectrum.

## 3. The phantom

### 3.1. Physical structured phantom

**Fig. 2:**
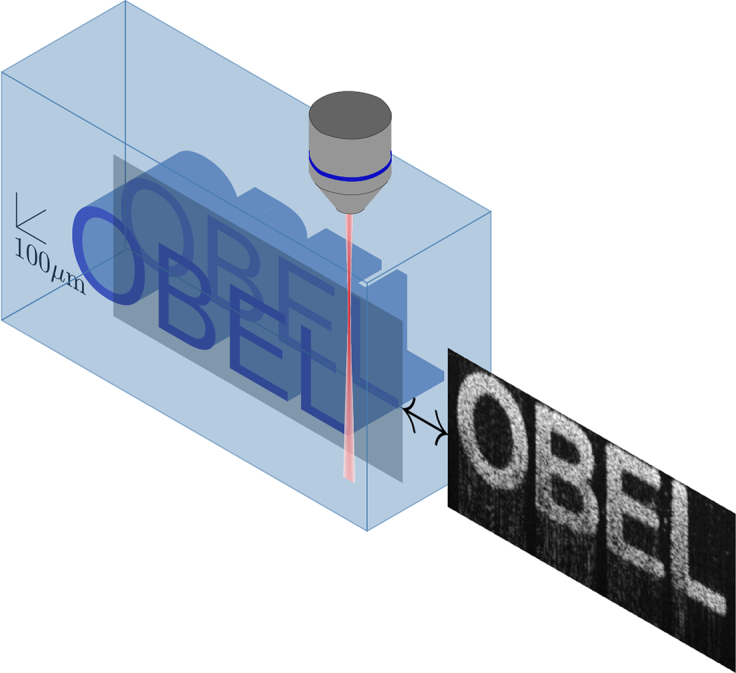
Schematic representation of the OBEL phantom, showing its orientation relative to the OCT objective (left) along with a typical B-scan of the phantom.

The physical, structured, OCT imaging phantom was developed and reported previously by Curatolo *et al.* [22]. We give only an outline of the details of the phantom here. Full details are available in the original publication. The phantom is three-dimensional and contains structured regions with a well defined scattering coefficient. It is designed such that B-scan images of the phantom reveal the letters “OBEL”, as is depicted schematically in Fig. 2. In particular, each letter is represented by volumes with a controlled scattering coefficient. These scattering volumes are created by dispersing a desired concentration of TiO_2_ particles (Sigma-Aldrich) into a two-part polydimethylsiloxane (PDMS) silicone (Dow Corning, Sylgard® 184 silicone elastomer). When the phantom used in this study was designed, the TiO_2_ spheres were assumed to have a diameter of 1*μ*m and a refractive index of 2.488 (at the design wavelength of *λ*_0_ = 1300nm), corresponding to the anatase form of TiO_2_, and were embedded within the letters at a concentration of 4.5×10^−3^ particles per cubic micron. In this study, however, the refractive index of the rutile form (2.609) was used as it predicts a lower scattering coefficient, closer to that observed in the experimental OCT images. Since performing the simulations for this study, we have found that the refractive index data on TiO_2_ is not as definitive as we had originally thought. In particular, we found that the above refractive indices are for a wavelength of 589nm and that both the anatase and rutile forms are uniaxially birefringent [23]. There thus remains significant uncertainty regarding the refractive index of the TiO_2_ particles used in the phantom. The silicone embedding medium was assumed to have a refractive index at *λ*_0_ of 1.42 which was obtained by experimental measurement using an OCT system and assuming that the group refractive index of the silicone provides a good estimate of its phase refractive index. This results in a scattering coefficient, calculated using Mie theory [24], at the design wavelength of 10.6 mm^−1^. The letters themselves are created using a deep-feature master, produced using UV photolithography, which is used as a mold. The mixture of silicone and TiO_2_ spheres is first introduced to this mold to create the letters. Once set and removed from the mold, the letters are embedded in silicone without TiO_2_ spheres. The phantom is thus useful for replicating many features of OCT image formation observed in tissue. The phantom is imaged from above the letters as is depicted in Fig. 2, along with a typical B-scan.

### 3.2. Numerical structured phantom

**Fig. 3:**
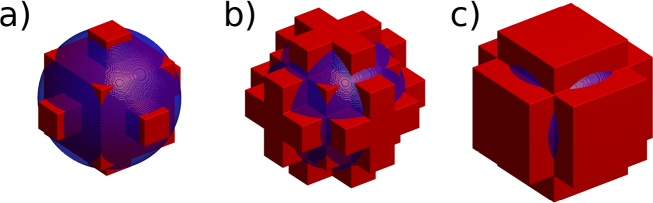
Three different discrete approximations to a sphere of diameter 1*μ*m using cubic elements of width *λ*_0_/6 (red) with a rendering of an ideal sphere of diameter 1*μ*m (blue) superimposed. We refer to a), b) and c) as discretisations 1, 2 and 3, respectively.

There is an inherent challenge with representing microscopic spherical scatterers using cubic cells with size comparable with that of scatterers. The primary advantage of the PSTD method is that it allows field quantities to be sampled at near the Nyquist rate, thus allowing large sample volumes to be modelled. The problem with this is that scatterers that contribute to image formation in OCT typically possess shapes which cannot be accurately represented on typical computational grids. For example, in the current work we considered a central wavelength of *λ*_0_ = 1300nm and an isotropic PSTD cell size of *λ*_0_/6. Three possible discrete approximations to such a sphere are shown in Fig. 3 along with the ideal sphere included as a reference. Note that the smooth surface of the sphere cannot be well represented on the computational grid. This problem is related to the problem of subpixel smoothing that has been the subject of much prior work in the field of FDTD modelling (see [25] for a good review of this subject). These subpixel smoothing approaches, however, require an anisotropic PSTD formulation which increases both the computer memory requirement and computational complexity of the PSTD model. It is for these reasons that we have sought an alternative solution to the accurate representation of scatterers with sizes comparable with that of the grid dimension.

**Fig. 4:**
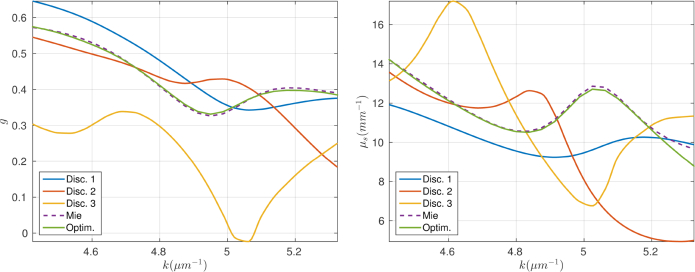
Plots of *g* (left) and *μ_s_* (right) within the OCT system’s spectrum for discretisations 1, 2 and 3 and the optimized design.

It is thus not surprising that the scattering properties of these discretised scatterers differ significantly from that of a sphere as is shown in Fig. 4. The plots of *μ_s_* in Fig. 4 have been obtained by calculating the scattering cross-section of each scatterer, using the PSTD method, and assuming a scatterer concentration equal to the design concentration used to make the physical phantom. The asymmetry parameter, *g*, for each scatterer is also plotted in Fig. 4, which was calculated by propagating the field scattered by each scatterer to a reference sphere in the far-field [26] allowing *g* to be calculated from first principles [24].

**Fig. 5:**
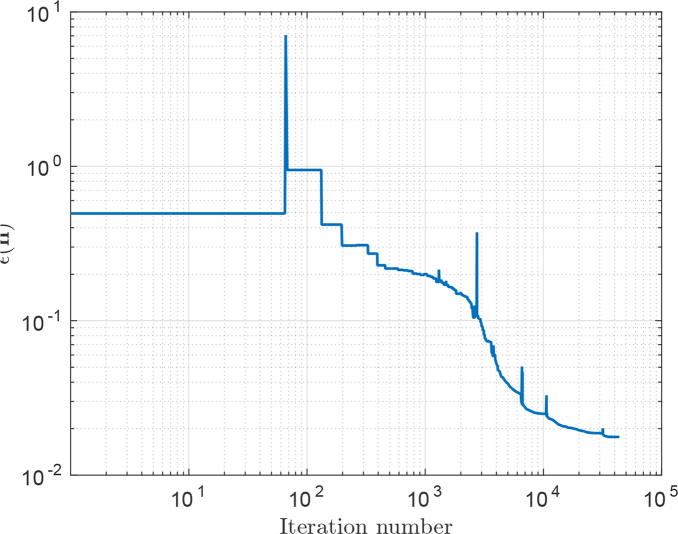
Plot of *∊*(***n***) versus the number of PSTD simulations executed during execution of optimization algorithm.

**Fig. 6:**
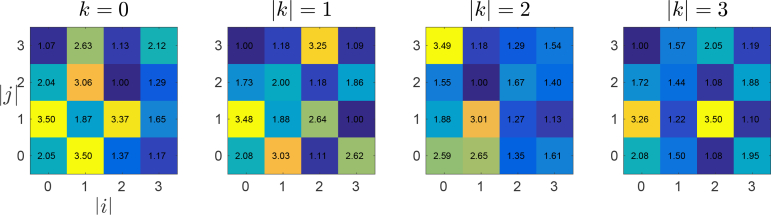
The optimized scatterer design, ***n***_opt_, at non-negative PSTD cell indices. The complete scatterer design can be obtained by noting that the refractive index of cell (*i*, *j*, *k*) is equal to that of (|*i*|, |*j*|, |*k*|).

An optimization problem was solved to find a scatterer with with three-dimensional scattering properties close to that of the TiO_2_ spheres which will thus also result in scattering cross-section and asymmetry parameter close to that of the TiO_2_ spheres. This was done by allowing the refractive indices of the PSTD cells filling a cube, which we denote the bounding cube, of side seven cells to vary between 1 and 3.5. The number of degrees of freedom was reduced from 7^3^ to 4^3^ by enforcing mirror symmetry about each orthogonal plane of PSTD cells bisecting the bounding cube. The optimization was performed iteratively by defining a mismatch function on a closed cubic reference surface composed of triangular facets defined by the set *F* which indexes a set of vertices *V*. We can thus denote a facet by *ijk* ∈ *F* with vertices *i*, *j*, *k* ∈ *V*. This allows for the definition of functions to compute the area and unit outward surface normal of a facet as *A_ijk_* and ${\hat{v}}_{\text{ijk}}$ , respectively. We also define the time-averaged Poynting vectors at vertex *i* for the discrete and Mie cases as ***S̃****_i_*(***n***, *λ*) and ***S****_i_*(*λ*), respectively, where ***n*** is a vector of 4^3^ unknown refractive index values. The mismatch function is thus defined as:

(8) $$∊(n)=\sum_{m=1}^{N_{\lambda }}S(\lambda_{m})\sqrt{\frac{\sum_{\forall ijk\in F}{\left|\frac{{\hat{\nu }}_{ijk}A_{ijk}}{3}\cdot \sum_{l=i,j,k}\left({\tilde{S}}_{l}(n,\lambda_{m})-S_{l}(\lambda_{m})\right)\right|}^{2}}{\sum_{\forall ijk\in F}{\left|\frac{{\hat{\nu }}_{ijk}A_{ijk}}{3}\cdot \sum_{l=i,j,k}S_{l}(\lambda_{m})\right|}^{2}}}$$

where the spectrum is sampled at discrete wavelengths *λ_m_*. We note that Eq. (8) is minimised when the spatial distribution of time-averaged energy flux, crossing the reference surface, of the discrete and Mie cases are in close agreement across the entire spectrum. In fact, if the reference surface were a sphere at infinity, this would amount to finding a discrete scatterer that matches the phase function of the target sphere across the spectrum. The close match between *μ_s_* and *g* for the discrete and Mie cases thus occurs as a result of matching the spatial distribution of time-averaged energy flux crossing the reference surface rather than being matched directly. It is necessary to execute a PSTD simulation to evaluate *∊*(***n***) for each ***n***. The refractive index distribution ***n***_opt_ was found using the MATLAB function fminsearch which resulted in a final value for *∊*(***n***_opt_) of 0.017. The fminsearch optimization started with an initial scatterer as shown in Fig. 3a) with the refractive index of the scattering cells set to 2.609. A plot of *∊*(***n***) versus the number of PSTD simulations is shown in Fig. 5. Just under 44000 PSTD simulations were executed taking approximately 13 days of wall-clock time on a workstation with dual Intel Xeon E5-2650 processors. We note that whilst techniques exist to significantly reduce the time taken to find such an optimum, these techniques were not employed. The optimized scatterer design is depicted in Fig. 6 in terms of PSTD grid indices, (*i*, *j*, *k*), where the scatterer was centred upon grid location (0, 0, 0). Due to the symmetry enforced on the structure of the scatterer, the scatterers refractive index structure is displayed for non-negative grid indices since the refractive index of cell (*i*, *j*, *k*) is identical to that of cell (|*i*|, |*j*|, |*k*|).

Figure 4 contains plots of both *μ_s_* and *g* for the optimized scatterer design. The plots show that both *μ_s_* and *g* agree closely with the ideal sphere calculated using Mie theory, across the spectrum. The most significant difference between the optimized scatterer and ideal sphere occurs for *μ_s_* at the highest wavenumber. This divergence was allowed by the mismatch function in Eq. (8) which weighted the mismatch between time-averaged Poynting vector flux according to the effective spectrum of the system.

## 4. Results

### 4.1. Optical system specifications

A Thorlabs Telesto-II spectral domain OCT system with an LSM03 objective was used to obtain the experimental images presented in this paper. Consultation with Thorlabs revealed that the optical system used in the Telesto-II is well represented by that shown in Fig. 1 with *f*_1_ = 25mm, *f*_2_ = 36mm, *R_a_* = 3.5mm and single mode optical fiber with a mode field diameter of 9.2*μ*m. The effective spectrum which takes into account the bandwidth of the light source and the spectrometer characteristics spans from 1180nm to 1420nm with a resulting central wavelength of 1300nm. This was measured on the system employed prior to shipping and could have changed over time, however, it is unlikely that any change will significantly change the outcome of this comparison. The Telesto-II has a spectrometer with 2048 pixels and this is how many wave numbers were calculated in each simulation. A Hanning window was applied to both the experimental and simulated data prior to reconstructing the OCT A-scans.

### 4.2. Point spread function phantom

**Fig. 7:**
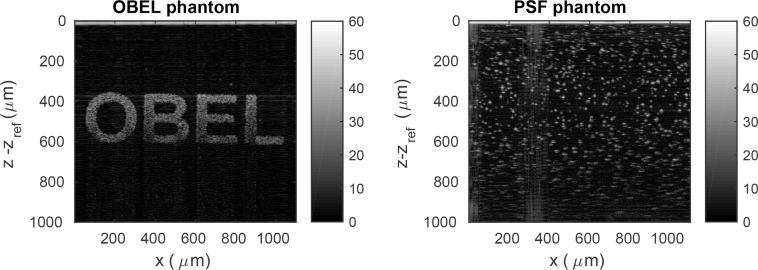
Comparison of B-scans of the OBEL and PSF phantoms displayed on an SNR scale ranging from 0 to 60 dB.

A point spread function (PSF) phantom manufactured by the National Physics Laboratory [27] was imaged under the same conditions as the OBEL phantom in order to confirm the performance of the OCT system. The PSF phantom is fabricated by embedding red iron oxide spheroidal nanoparticles (07674, Polysciences, Incorporated, USA), with a predominant diameter of 400 nm [27], into a two-part polyurethane resin (DR006, Atlas Polymers, UK). It was checked that these nanoparticles were suitable for PSF measurement using an OCT system such as used in this study [28]. The nanoparticles are embedded at a low density such that they appear as isolated scatterers in the OCT image. A measured B-scan of the PSF phantom is shown alongside a B-scan of the OBEL phantom in Fig. 7. The PSF phantom is useful for verifying that the model predicts the correct PSF and also for checking where the OCT system’s objective is focussed.

**Fig. 8:**
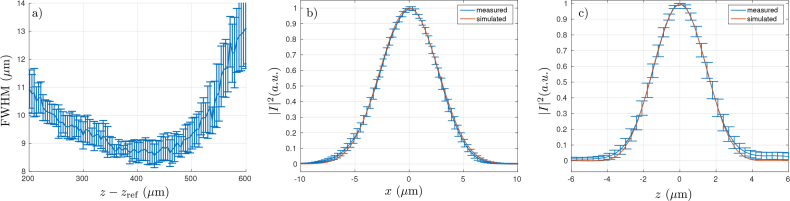
Plots of data extracted from the PSF phantom. a) Shows the average lateral FWHM of point objects as a function of axial location. The error bars span two standard deviations. b) Shows the average of measured lateral PSFs located within the region |*z* − *z*_ref_ − 400*μm*| < 20*μm* with error bars spanning two-standard deviations along with the simulated in-focus lateral PSF. c) Shows the average of axial PSFs of the same point objects considered in b) along with the simulated axial PSF. The error bars span a total of two standard deviations.

In order to check the location of focus of the beam, the full width at half maximum (FWHM) of the lateral PSFs were extracted from various axial locations from within the PSF phantom and are plotted in Fig. 8a). This was performed by considering an axial range of total depth 40*μ*m centered upon the sampled values of *z* − *z*_ref_. The measured PSFs were automatically selected from the axially truncated C-scan. This was done by first calculating a histogram of all C-scan pixel values and obtaining the upper 0.05% of pixel magnitudes. The MATLAB function bwlabel was then used to identify connected volumes of pixels in the upper 0.05% of pixel magnitudes. From each connected volume of pixels, the maximum was found and this maximum was assumed to be near the centre of an isolated PSF. A Gaussian function was then fitted in the *x*- and *z*-directions about the determined centre pixel. This fitting step performed three functions. First, the quality of fit was used to eliminate PSFs which were in fact due to a combination of two or more scatterers. Second, the sub-pixel alignment of the PSF was determined. Third, the FWHM of lateral PSFs was obtained as a function of *z* − *z*_ref_, which are plotted in Fig. 8a). This plot allows us to conclude that the OCT system was focussed just below *z* − *z*_ref_ = 400*μm*, which from the corresponding phantom plot is in the vicinity of the top of the letters “OBEL”.

Whilst acquiring the lateral FWHM data, the lateral and axial PSFs in the range |*z* − *z*_ref_ − 400*μm* | < 20*μm* were acquired from the image of the PSF phantom. This data is obtained by re-sampling each measured PSF onto common axial and lateral sample lines, as is necessary due to the sub-pixel misalignment of each PSF. Interpolation was performed using the piecewise cubic Hermite interpolating polynomial (i.e. “pchip”) provided by MATLAB. The simulated lateral PSF was calculated firstly by calculating the form of the incident focussed field at the focus of the computational space. This was done using the Debye-Wolf integral as used to calculate ***e***_ill_(***r***_3_, *k*_0_) as discussed in Sec. 2.1. It has already been shown that the general PSTD-based imaging model accurately simulates PSFs [1] and so this work is not reproduced here. The simulated axial PSF was found by evaluating Eq. (7) with *ñ*(*k*) set to unity, i.e., the Fourier transform of the spectrum was evaluated.

### 4.3. Structured phantom

**Fig. 9:**
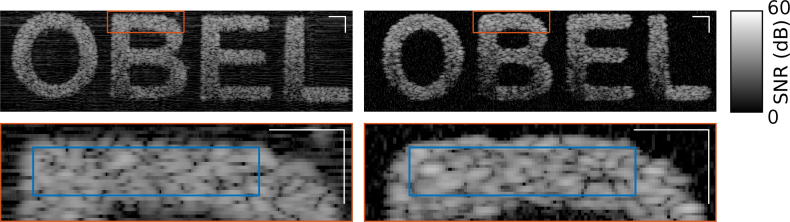
Comparison between experimental (left) and simulated (right) images of the OBEL phantom. The lower image in each column is an expanded view of the region bounded by a red rectangle in the full image of the same column. The white right-angle in each image denotes 50*μ*m. The blue rectangle in the expanded images denotes the region used to calculate the autocovariance plots in Fig. 10. The axial dimension is scaled to physical distance in both cases.

Experimental and simulated images of the OBEL phantom are shown in Fig. 9 on a logarithmic signal to noise ratio (SNR) scale. A total of 544 A-scans were calculated on compute nodes each containing two Intel Xeon E5-2690v4 (Broadwell) 2.6GHz processors. Each A-scan took approximately 17 hours wall-clock time to compute resulting in a total wall-clock time of 385 days. Each simulation required 10.2 Gb of random access memory. Pixelation is noticeable in the lateral direction of the simulated case since the lateral pixel size was made larger than that of the experimental image in order to reduce computation time, since each simulated A-scan represents an independent simulation. The white right-angle scale bars denote a dimension of 50*μm*. A region of the “B” in the full phantom images is expanded in the lower rows to enable the nature of the speckle pattern to be better appreciated. The images in Fig. 9 reveal some differences between the experimental and simulated cases. The most striking of these is the higher attenuation noticeable along the length of the letters in the simulated images, parallel to the OCT system’s optical axis. We consider this further in Sec. 4.3.1. There is also a small difference between the shapes of the letters. We investigated this by comparing a profilometry image of the physical phantom immediately after casting [22], from which the numerical phantom was designed. It was found that whilst the simulated image faithfully replicated the shape of the profilometry image, the experimental image did not. In particular, whilst the spacing between letters was consistent with the profilometry image, the letter shapes in the experimental image had undergone subtle change. We then investigated several B-scans such as are depicted in Fig. 9 and found that the letter shapes exhibited variation amongst different B-scans. This subtle deformation most likely arises when the features (i.e., the letters “OBEL”) are finally embedded in silicone.

**Fig. 10:**
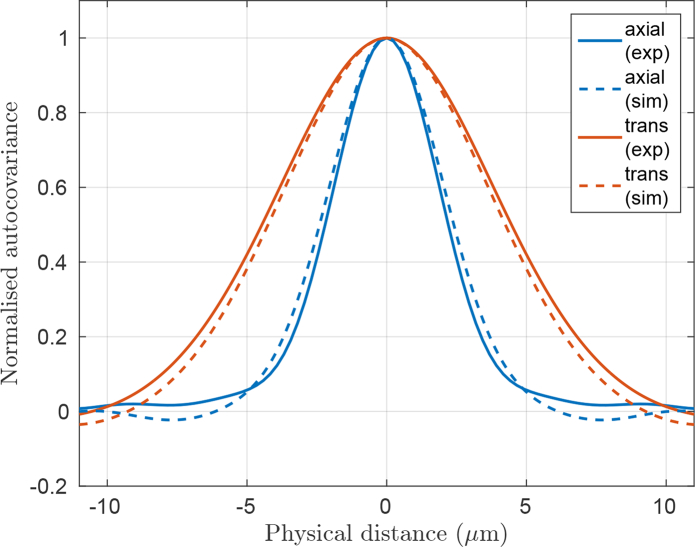
Plots of normalized autocovariance along the axial and transverse directions, for simulated and experimental phantoms.

Whilst the lower row of images in Fig. 9 suggests that the speckle size in the simulated and experimental images are consistent, we employed a quantitative technique to objectively verify this. We plotted the normalized autocovariance [29] of regions bounded by the blue rectangle in the expanded images Fig. 9. The resulting plots of autocovariance along the axial and lateral directions are shown in Fig. 10. The full width at half maximum (FWHM) of the experimental autocovariance plots, averaged across all available B-scans, was 4.8*μ*m±0.4*μ*m and 9.3*μ*m±1.5*μ*m in the axial and lateral directions, respectively, where the uncertainty corresponds to one standard deviation. This compared with results from the simulated autocovariance plots of 5.2*μ*m and 8.5*μ*m, respectively, thus being consistent with the experimental values.

**Fig. 11:**
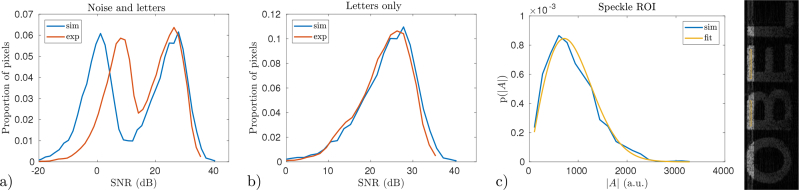
Histograms of pixel values for the experimental and simulated images. Pixels were taken from the top 35*μ*m of the image occupied by the letters, as is illustrated in the right image. a) Shows the histogram when the entire rectangular region depicted in the right image is considered. b) Shows the histogram when only pixels falling within the stencil of the letters are considered and plot c) shows an amplitude probability distribution (p(|*A*|)) of the noise-free simulated pixels from within the ROI (indicated by yellow boxes), along with a fitted Rayleigh distribution.

The histogram of pixel values from the experimental and simulated images provides for another way to compare the two images. We consider the histograms for a rectangular region of interest as wide as the lateral extent of the images and 35*μ*m deep as depicted in the image of Fig. 11. The region of interest was set to intersect the top-most horizontal letter features. Plot a) of Fig. 11 shows the histogram for all pixels within the rectangular region of interest. This histogram contains two peaks corresponding to the letters and noise, respectively. The noise in the experimental image has a higher mean than in the simulated case. The noise in this region must also be greater than the noise in the region originally used to normalize the experimental image. The noise in the simulated case is centred on 0dB SNR as expected. When only the pixels falling within the stencil of the letters are considered, as shown in plot b) of Fig. 11, the agreement between theory and experiment is very close. Plot c) of Fig. 11 shows that the noise-free pixel values taken from a shallow axial range of the simulated image, away from the edges of the letters, closely follow a Rayleigh distribution, as expected. We emphasize here that neither the simulated or experimental data were manipulated to equalize the histograms, the value of *η* was chosen, as discussed in Sec. 2.4, to match the mean SNR in a region of interest at the top of the letter “B”.

#### 4.3.1. Analysis of scattering coefficient

**Fig. 12:**
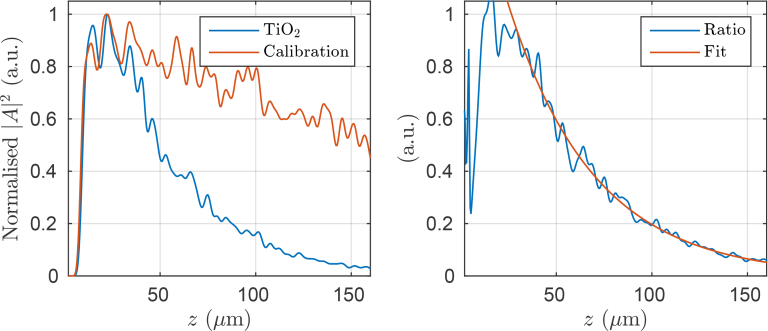
The left plot contains averaged A-scan intensities for different arrangements of the modelled TiO_2_ scatterers and particles with a low scattering cross-section in order to produce a calibration A-scan. The right plot presents a plot of the ratio between the two plots from the left axis along with an exponential fit to this data.

We performed some additional simulations in order to investigate why the experimental and simulated images result in significantly different scattering coefficients in the letters of the “OBEL” phantom. In particular, we calculated 150 A-scans for differing random arrangements of the optimized scatterer at the design concentration of the physical phantom. Similarly, we calculated 300 A-scans for differing random arrangements of scatterers with a very low scattering cross-section by using the scatterer displayed in Fig. 3 with its scattering cells set to have a refractive index 1.421, resulting in a very small refractive index contrast with surrounding medium which had refractive index 1.42, and thus a negligibly small scattering coefficient. The resulting intensity averaged A-scans are plotted in the left plot of Fig. 12. The plots have been independently normalized and are displayed using arbitrary units. The low scattering average A-scan was obtained in order to calculate the so-called confocal function which takes account of the attenuation in OCT signal with depth due to the axial PSF that the OCT system would have if it were operated as a coherent scanning microscope, i.e., coherence gating was not employed. We note that even though 150 and 300 intensity A-scans have been averaged in the high and low scattering cases, respectively, significant speckle still remains present in the plots.

By dividing the high scattering average A-scan by the low scattering average A-scan, a plot of the sample induced attenuation is obtained as is shown in the right hand plot of Fig. 12. By assuming that single scattering dominates the A-scans to the depth considered in Fig. 12, an exponential fit of the form *I* = *I*_0_ exp(−2*μ_s_*(*z* − *z*_0_)) was then obtained and is also plotted in the right axis of Fig. 12. We note that this kind of analysis continues to be the subject of much interest and we thus direct readers to works such as by Almasian *et al.* [30], where this approach is treated rigorously. The result of this analysis is an estimate for the scattering coefficient for the optimized scatterer design at the design concentration of *μ_s_* = 11.1 ± 0.1mm^−1^. The uncertainty was estimated using the standard deviation of each ratio data point of Fig. 12. In particular, many fits were performed where different random perturbations, taken from the known uncertainty distribution, were added to each point in the data to be fitted. The uncertainty of ±0.1mm^−1^ is the standard deviation of the resulting distribution of *μ_s_* values. This compares with the design value of 10.6mm^−1^ at the design wavelength, however, when the scattering coefficient is calculated by averaging the scattering-cross section over the entire spectrum, weighted by *S*(*k*), a value of 11.4mm^−1^ is obtained. A further investigation of this is beyond the scope of this study, however, we believe that the scattering coefficient retrieved from the average A-scans is consistent with that expected from theory given the design parameters of the phantom.

## 5. Discussion and conclusion

Significant progress has been made recently in being able to simulate image formation in OCT, for sample volumes of practical importance, with a high degree of realism. Despite this, however, there remains a trade-off between the volume of sample that can be modelled and the spatial resolution at which sample structure can be represented. This trade-off is accentuated by the result in this paper which shows how the microstructure of scatterers strongly influences both its scattering cross-section and asymmetry parameter. This paper demonstrates that optimized scatterer design offers a way to overcome the limit of simulating image formation for scatterers with fine microstructure embedded within a physically large sample volume. It is important to note, however, that we have not considered an inverse scattering problem. In particular, the oscillatory nature of the resulting optimized scatterer design suggests that the solution we obtained is not very stable, as would be desired when solving an inverse scattering problem. Rather, we sought to find a refractive index distribution, not necessarily one that is unique or stable, that replicates the scattering properties of the scatterer that we wish to emulate.

In performing this work we have provided experimental validation of the three-dimensional OCT image formation model for two phantoms, one for which the Born approximation is valid and one for which it is not. We have shown that the model reproduces the point spread function of an experimental system. We have also shown that, in the more complex “OBEL” phantom, the model predicts a speckle size that is in agreement with experiment. The model also results in a histogram of pixel values within the signal region of the phantom image that agrees with that of the experimental case. We emphasize here that the numerical model was based entirely on the design parameters of the physical phantom. In particular, none of the parameters of the numerical model were modified in order to achieve better agreement between simulation and experiment, other than the noise parameter *η* which is necessarily dependent upon an experimentally acquired image. We also emphasize here that as this model is based on a full wave simulation of light propagation, it implicitly includes phenomena such as multiple scattering and dependent scattering.

Despite the general agreement between theory and experiment, the simulated and experimental images diverged in the degree of attenuation along the axial direction of the letters in the “OBEL” phantom. We believe that this is, however, due to uncertainty in the properties of the experimental phantom rather than a shortcoming of the simulation. In particular, the TiO_2_ particles were acquired in powder form with a mean diameter of 1*μ*m. The size distribution of these particles and how spherical they were was never established. It has also been shown that PDMS can acquire refractive index inhomogeneity at interfaces with other materials and PDMS itself (i.e., at a PDMS-PDMS interface) [31]. Whilst this could also explain a deviation in the observed scattering coefficient in the “OBEL” phantom, it may also describe the origin of the vertical line type feature running along the left hand vertical edges of the “B”, “E” and “L” letters in the experimental image. It is thus not surprising that there is some divergence between the simulated and experimental images in this work.

We anticipate that this work will enable highly realistic simulation in a range of OCT applications. For example, biological tissues are often characterised experimentally in terms of scattering coefficient and asymmetry parameter. Thus, rather than try to represent biological tissues in terms of a microscopic refractive index distribution, which cannot be measured, optimized scatterers can be derived which represent the average scattering properties of tissue. This will allow various tissues to be arranged in a meaningful way in order to investigate biomedical applications of OCT. The model will also be very powerful in investigating quantitative techniques such as parametric imaging [30] which are rapidly emerging.

## References

[1] Munro P. R. T., “Three-dimensional full wave model of image formation in optical coherence tomography,” Opt. Express 24, 27016–27031 (2016). 10.1364/OE.24.027016 27857429

[2] Brenner T. Reitzle D. Kienle A., “Optical coherence tomography images simulated with an analytical solution of Maxwell’s equations for cylinder scattering,” J. Biomed. Opt. 21, 45001 (2016). 10.1117/1.JBO.21.4.045001 27032336

[3] Munro P. R. T. Curatolo A. Sampson D. D., “Full wave model of image formation in optical coherence tomography applicable to general samples,” Opt. Express 23, 2541–2556 (2015). 10.1364/OE.23.002541 25836119

[4] Krüger B. Brenner T. Kienle A., “Solution of the inhomogeneous Maxwell’s equations using a Born series,” Opt. Express 25, 25165–25182 (2017). 10.1364/OE.25.025165 29041187

[5] Wilson T. Sheppard C., Theory and Practice of Scanning Optical Microscopy (Academic, 1984).

[6] Ralston T. S. Marks D. L. Carney P. S. Boppart S. A., “Inverse scattering for optical coherence tomography,” J. Opt. Soc. Am. A 23, 1027–1037 (2006). 10.1364/JOSAA.23.001027 16642179

[7] Coupland J. M. Lobera J., “Holography, tomography and 3D microscopy as linear filtering operations,” Meas. Sci. Technol. 19, 074012 (2008). 10.1088/0957-0233/19/7/074012

[8] Villiger M. Lasser T., “Image formation and tomogram reconstruction in optical coherence tomography,” J. Opt. Soc. Am. A 27, 2216–2228 (2010). 10.1364/JOSAA.27.002216 20922012

[9] Schmitt J. M. Knuttel A., “Model of optical coherence tomography of heterogeneous tissue,” J. Opt. Soc. Am. A 14, 1231–1242 (1997). 10.1364/JOSAA.14.001231

[10] Thrane L. Yura H. T. Andersen P. E., “Analysis of optical coherence tomography systems based on the extended Huygens-Fresnel principle,” J. Opt. Soc. Am. A 17, 484–490 (2000). 10.1364/JOSAA.17.000484 10708029

[11] Pan Y. Birngruber R. Rosperich J. Engelhardt R., “Low-coherence optical tomography in turbid tissue: theoretical analysis,” Appl. Opt. 34, 6564–6574 (1995). 10.1364/AO.34.006564 21060511

[12] Smithies D. J. Lindmo T. Chen Z. Nelson J. S. Milner T. E., “Signal attenuation and localization in optical coherence tomography studied by Monte Carlo simulation,” Phys. Med. Biol. 43, 3025–3044 (1998). 10.1088/0031-9155/43/10/024 9814533

[13] Yao G. Wang L. V., “Monte Carlo simulation of an optical coherence tomography signal in homogeneous turbid media,” Phys. Med. Biol. 44, 2307–2320 (1999). 10.1088/0031-9155/44/9/316 10495123

[14] Tycho A. Jorgensen T. M. Yura H. T. Andersen P. E., “Derivation of a Monte Carlo method for modeling heterodyne detection in optical coherence tomography systems,” Appl. Opt. 41, 6676–6691 (2002). 10.1364/AO.41.006676 12412659

[15] Lu Q. Gan X. Gu M. Luo Q., “Monte Carlo modeling of optical coherence tomography imaging through turbid media,” Appl. Opt. 43, 1628–1637 (2004). 10.1364/AO.43.001628 15046164

[16] Meglinski I. Kirillin M. Kuzmin V. Myllyla R., “Simulation of polarization-sensitive optical coherence tomography images by a Monte Carlo method,” Opt. Lett. 33, 1581–1583 (2008). 10.1364/OL.33.001581 18628804

[17] Ossowski P. Wojtkowski M. Munro P. R. T., “Classification of biological micro-objects using optical coherence tomography: in silico study,” Biomed. Opt. Express 8, 3606–3626 (2017). 10.1364/BOE.8.003606 28856039 PMC5560829

[18] Richards B. Wolf E., “Electromagnetic diffraction in optical systems ii. structure of the image field in an aplanatic system,” Proc. Roy. Soc. A 253, 358–379 (1959). 10.1098/rspa.1959.0200

[19] Munro P. R. T., “Exploiting data redundancy in computational optical imaging,” Opt. Express 23, 30603–30617 (2015). 10.1364/OE.23.030603 26698693

[20] Munro P. R. T. Engelke D. Sampson D. D., “A compact source condition for modelling focused fields using the pseudospectral time-domain method,” Opt. Express 22, 5599–5613 (2014). 10.1364/OE.22.005599 24663901

[21] Munro P. R. T. Török P., “Calculation of the image of an arbitrary vectorial electromagnetic field,” Opt. Express 15, 9293–9307 (2007). 10.1364/OE.15.009293 19547272

[22] Curatolo A. Kennedy B. F. Sampson D. D., “Structured three-dimensional optical phantom for optical coherence tomography,” Opt. Express 19, 19480–19485 (2011). 10.1364/OE.19.019480 21996888

[23] Rumble J.R., “CRC Handbook of Chemistry and Physics” (CRC, 2018)

[24] Bohren C. Huffman D., Absorption and Scattering of Light by Small Particles (Wiley Interscience, 1983).

[25] Oskooi A. Johnson S. G., “Accurate FDTD Simulation of Discontinuous Materials by Subpixel Smoothing,” in Advances in FDTD Computational Electrodynamics: Photonics and Nanotechnology, Taflove A. Oskooi A. Johnson S. G., eds. (Artech House Antennas and Propagation Series, 2013), pp. 133–148.

[26] Török P. Munro P. R. T. Kriezis E. E., “Rigorous near- to far-field transformation for vectorial diffraction calculations and its numerical implementation,” J. Opt. Soc. Am. A 23, 713–722 (2006). 10.1364/JOSAA.23.000713 16539070

[27] Woolliams P. D. Ferguson R. A. Hart C. Grimwood A. Tomlins P. H., “Spatially deconvolved optical coherence tomography,” Appl. Opt. 49, 2014–2021 (2010). 10.1364/AO.49.002014 20389999

[28] Tomlins P. H. Ferguson R. A. Hart C. Woolliams P. D., “Point-spread function phantoms for optical coherence tomography,” NPL Report OP2 (National Physical Laboratory, 2009).

[29] Ossowski P. Raiter-Smiljanic A. Szkulmowska A. Bukowska D. Wiese M. Derzsi L. Eljaszewicz A. Garstecki P. Wojtkowski M., “Differentiation of morphotic elements in human blood using optical coherence tomography and a microfluidic setup,” Opt. Express 23, 27724–27738 (2015). 10.1364/OE.23.027724 26480435

[30] Almasian M. Bosschaart N. van Leeuwen T. G. Faber D. J., “Validation of quantitative attenuation and backscattering coefficient measurements by optical coherence tomography in the concentration-dependent and multiple scattering regime,” J. Biomed. Opt. 20, 121314 (2015). 10.1117/1.JBO.20.12.121314 26720868

[31] Martinček I. Turek I. Tarjányi N., “Effect of boundary on refractive index of PDMS,” Opt. Mater. Express 4, 1997–2005 (2014). 10.1364/OME.4.001997

